# Role of human dural fibroblasts in the angiogenic responses of human endothelial cells: An in vitro dural model and the application of lab‐on‐a‐chip for EDAS

**DOI:** 10.1002/btm2.10589

**Published:** 2023-08-19

**Authors:** Woo‐Keun Kwon, Chang‐Min Yoo, Jang Hun Kim, Tae‐Won Kim, An‐Gi Kim, Min‐Ho Hwang, Hyuk Choi

**Affiliations:** ^1^ Department of Neurosurgery, Korea University Guro Hospital Korea University College of Medicine Seoul South Korea; ^2^ Department of Medical Sciences, Graduate School of Medicine Korea University Seoul South Korea; ^3^ Department of Neurosurgery, Korea University Anam Hospital Korea University College of Medicine Seoul South Korea

**Keywords:** angiogenesis, dura mater, Encephaloduroarteriosynangiosis, inflammation, Moyamoya disease

## Abstract

Encephaloduroarteriosynangiosis (EDAS), an indirect anastomosis procedure, is widely accepted as a primary treatment for moyamoya disease (MMD) to improve collateral blood flow. During surgical intervention, dural fibroblasts (DuF) are thought to produce various proteins that create an angiogenic microenvironment. However, the biophysiological evidence supporting the angiogenic properties of this surgical technique has not been thoroughly elucidated. The purpose of these studies was to determine whether DuF releases pro‐angiogenic factors and chemokines and promotes angiogenic properties in human endothelial cells (ECs) under IL‐1β‐mediated wound conditions, which are expected to occur during the process of neo‐vascularization within the dura mater. Furthermore, a microfluidic chemotaxis platform was implemented to investigate the angiogenic activity of ECs in response to a reconstituted dura model. Transcriptome sequencing revealed that IL‐1β stimulation on DuF induced a significant upregulation of various pro‐angiogenic genes, including IL‐6, IL‐8, CCL‐2, CCL‐5, SMOC‐1, and SCG‐2 (*p* < 0.05). Moreover, compared to ECs cultured in naïve media or naïve DuF media, those exposed to IL‐1β‐DuF conditioned media expressed higher mRNA and protein levels of these pro‐angiogenic factors (*p* < 0.001). ECs co‐cultured with IL‐1β‐DuF also exhibited considerable migration on the microfluidic chemotaxis platform. Furthermore, the chemotactic effects on the ECs were reduced upon neutralization of IL‐8 or inhibition of NF‐κB signaling. Our findings demonstrate that IL‐1β‐DuFs release factors that activate and enhance the angiogenic properties of ECs. These results suggest a potential interaction between DuF and ECs following EDAS for MMD, and these components could be targeted for the development of therapeutic biomarkers.

**Translational Impact Statement**

Moyamoya disease is a neurovascular steno‐occlusive disorder, and surgical revascularization techniques such as encephaloduroarteriosynangiosis (EDAS) have been widely performed. However, little is known about its cellular and biological mechanisms. In our study, we have discovered that the interaction between dural fibroblasts and endothelial cells leads to enhanced angiogenic activities and significant migration of endothelial cells. This finding represents a significant advancement in our understanding of the molecular mechanisms involved in the interaction between dural fibroblasts and endothelial cells during the angiogenesis process following surgical intervention.

## 1 INTRODUCTION

Moyamoya disease (MMD) is a chronic cerebrovascular disorder characterized by progressive occlusion of the bilateral distal internal carotid arteries, which subsequently affects the major intracranial circulation and results in compensatory development of microvessels into the brain parenchyma. Altered vascular structures and intracranial circulation subsequently lead to catastrophic clinical presentations such as cerebral ischemia or hemorrhages.^1^, ^2^ Although the prevalence and incidence of this specific disease are extremely low, 18.1% and 4.3%, respectively, in 2013, MMD has become a significant public health problem considering its potential enormous personal/socioeconomic burden.^3^, ^4^ Encephaloduroarteriosynangiosis (EDAS) is an indirect vascular bypass method, which involves the transposition of a segment of a scalp artery onto the surface of the brain, which aims to improve collateral blood flow.^5^ It has been utilized and performed for MMDs for many years, in order to prevent secondary stroke or potential hemorrhagic insults in patients with MMD.^6^ EDAS surgically embeds a branch of the superficial temporal artery and the adjacent dura mater on the pial surface of the brain.^7^, ^8^ It is expected to eventually develop new vessels formed by pre‐existing endothelial cells, known as “synangiogsis,” resulting in the enhanced transport of oxygen and nutrients to deeper regions of the brain through those new vascular networks.^9^, ^10^ Although the clinical outcome of MMD has been well documented and numerous studies have been undertaken to explore optimal strategies for inducing therapeutic angiogenesis, the molecular mechanism and advanced therapeutic strategies for enhancing angiogenic properties in the EDAS procedure have not been thoroughly elucidated. While clinical evidence of the efficacy of EDAS for MMD excels, the lack of experimental biophysiological evidence of this technique remains a limitation of this widely accepted surgical procedure.

The meninges are a subset of connective tissues and comprise three major membrane layers that cover and protect the brain and spinal cord: the dura mater, which is the outermost meningeal layer residing just beneath the bone; arachnoid mater; and pia mater, which is located just above the brain.^11^, ^12^ Unlike the underlying pia or arachnoid mater, the dura mater is a highly vascularized collagenous membrane that mechanically protects the intracranial structures and modulates the meningeal immune response.^11^, ^13^ The dura mater is predominantly composed of dural fibroblasts (DuFs) widely spaced in a vast extracellular matrix (ECM) of collagen and fibronectin and has a large repertoire of immune sentinels, including dendritic cells, resident macrophages, neutrophils, T cells, and B cells.^14^, ^15^ DuFs are responsible for the regulation of neighboring blood vessels and immune cells through the production of cytokines, growth factors, and ECM‐modifying enzymes. DuF recruits and interact with immune cells in both healthy and inflammatory states. In particular, under inflammatory microenvironment induced by external and/or endogenous stimuli, DuF can potentially recruit immune cells to sites of injury by releasing chemokines and inflammatory mediators.^16^, ^17^ Infiltrating immune cells and/or resident cells such as fibroblasts express pro‐inflammatory cytokines such as interleukin‐1beta (IL‐1β), which is well known as a master regulator of the inflammatory response and wound healing. IL‐1β has several common functions, including chemoattraction of neutrophils, induction of angiogenic responses in endothelial cells, and production of various cytokines by fibroblasts in diverse tissues. Furthermore, IL‐1β promotes the activation and migration of fibroblasts, stimulating their transformation into myofibroblasts, which are key players in wound healing and scar formation.^18^, ^19^ However, the effects of phenotypic and genotypic changes in human DuF induced by IL‐1β stimulation are largely unknown. In addition, the effects of the angiogenic response on endothelial cells following intra/inter‐cellular signaling with human DuF have not been thoroughly elucidated.

Indirect bypass procedures such as EDAS are generally offered to patients with intracranial steno‐occlusive diseases such as MMD who suffer from symptoms or complications following progressive reduction in cerebral blood flow.^20^, ^21^ During the surgical procedure, the dura mater, which consists of human DuF, is incised and physically and/or chemically injured, and a consequent cascade of the inflammatory response and wound healing process by DuF follows accompanied by production of diverse inflammatory mediators and chemotactic factors. Additionally, the injured/incised dura mater is embedded on the surface of the brain parenchyma, and active interaction between the vascular structures of the brain and the dura mater is expected. This consideration corroborates the hypothesis that, under this inflammatory microenvironment, consequent interaction between DuF and vascular endothelial cells may occur and possibly contribute to neovascularization after surgical intervention with EDAS.

In the context mentioned above, it is crucial to develop an efficient biomimetic in vitro model that simulates the three‐dimensional microenvironment of the dura mater, including endothelium along with the cell‐to‐cell interactions. However, the currently available in vitro cell culture systems, such as the transwell platform, have limitations in recapitulating the biological characteristics due to their vertically arranged compartments. This arrangement makes it challenging to monitor and analyze cells in different focal planes in real time. Moreover, the 2D monolayer culture within the transwell lacks crucial ECM components such as collagen, which can significantly influence the genotypic/phenotypic expression of cells. Therefore, it is imperative to develop a more advanced in vitro model that can overcome these limitations.

One proposed solution is the development of a lab‐on‐a‐chip platform capable of replicating key components of tissue‐specific microenvironments. Lab‐on‐a‐chip platforms are considered more physiologically relevant compared to conventional 2D in vitro models since they provide a controlled spatiotemporal environment, including diffusive fluid and ECM‐encapsulated culturing, that facilitates cell‐to‐cell paracrine signaling or biomimetic cell response. Various lab‐on‐a‐chip models that mimic the pathogenesis of diverse organ‐based diseases, such as the liver, kidney, and lung have been investigated. However, the angiogenic response mediated by the interaction between the dura mater and endothelial cells, which is expected to occur during EDAS intervention, remains not fully understood.

In this context, we studied the effects of soluble factors derived from DuF induced by IL‐1β stimulation on human microvascular endothelial cells in order to elucidate the possible in vivo reaction during neovascularization after surgical intervention with EDAS. We hypothesized that DuF stimulated by IL‐1β would express inflammatory mediators, proangiogenic factors, and chemokines at both the gene and protein levels eventually acting as pro‐angiogenic stimulation to endothelial cells. Moreover, we developed a microfluidic platform to study the motility of endothelial cells in response to soluble factors derived from IL‐1β‐stimulated DuF under co‐culturing conditions.

## 2 RESULTS

### 2.1 IL‐1β stimulation of human DuF induces an inflammatory response by modulating the gene and protein expression of inflammatory mediators and by the preferential distribution of NF‐κB p65 protein into the nucleus

IL‐1β, a prototypical “proinflammatory cytokine,” is essential for cellular defense and repair in nearly all tissues. It plays a major role in the inflammatory response in brain tissue by modulating inflammatory mediators, angiogenic factors, and chemokines via the NF‐κB signaling pathway. In this study, to verify that IL‐1β stimulation induces an inflammatory response in human DuF, which is expected to occur during the EDAS surgical procedure via NF‐κB p65 protein translocation into the nucleus, the cells were treated with recombinant human IL‐1β (1 ng/mL) for 48 h. Afterward, the translocation of p65 protein was then captured at different times (0, 10, 30, and 60 min) using immunofluorescence microscopy.

**FIGURE 1:**
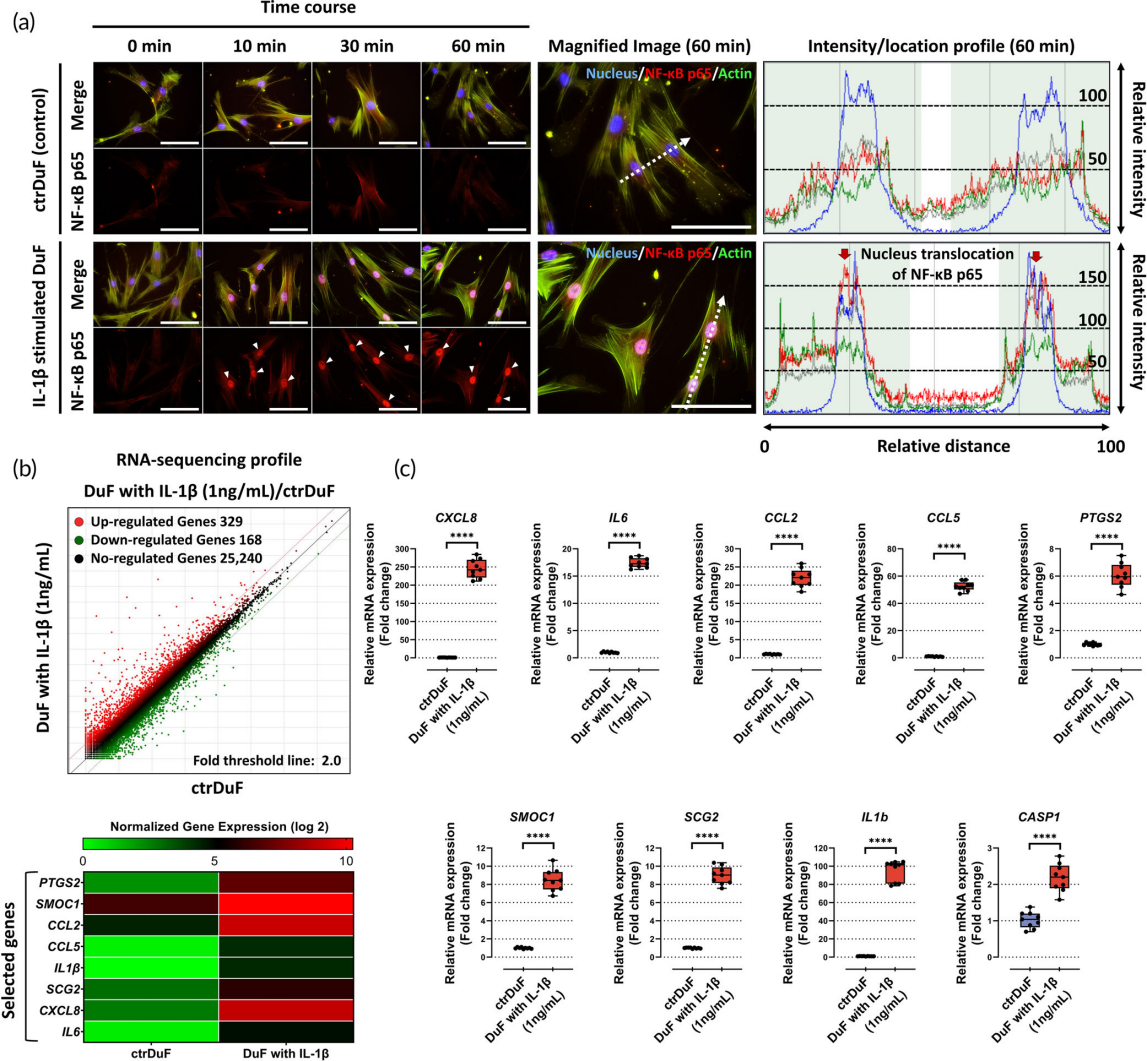
Activation of the NF‐κB signaling pathway and the expression of angiogenic genes and chemokines in IL‐1β‐stimulated DuF. (a) Fluorescence images and quantitative results show the translocation of NF‐κB p65 protein, which is a subunit of NF‐κB complex (p65/p50 complex), into nucleus in IL‐1β‐stimulated DuF. Red arrow indicates the preferential distribution of this protein in the nucleus. Its nucleus translocation in the nucleus functions as a transcription factor that induces the encoding of inflammatory and catabolic genes. Scale bar = 100 μm. (b) Using RNA‐seq analysis, the differentially expressed genes (DEGs) in DuF with or without IL‐1β are illustrated as a scatter plot. Normalized gene expression (log 2) > 4.0 and adjusted *p* value <0.05 were used as the cutoff to identify DEGs. Heatmaps show the expression of a class of pro‐angiogenic and chemotactic genes in different groups. Based on these results, nine genes were selected as target factors in this study. (c) qRT‐PCR analysis of the expression of the selected genes and *CASP1* in DuF with or without IL‐1β. The results show significantly higher expression of all genes and *CASP1* in IL‐1β‐stimulated DuF compared with those in naïve DuF. The values are reported as the mean ± standard error of nine independent experiments. ****p < 0.0001 compared to naïve DuF.

Fluorescence images and quantitative intensity revealed that in the presence of recombinant IL‐1β, the NF‐κB p65 protein was preferentially distributed in the nucleus rather than in the cytoplasm of human DuF at 10, 30, and 60 min, whereas in the absence of IL‐1β, it was present in the cytoplasm and showed a low fluorescence intensity (Figure 1a). These results indicate that IL‐1β stimulation promotes the translocation of NF‐κB p65 protein into the nucleus of human DuF, where it is associated with the inflammatory response by acting as a transcription factor.

Second, to obtain gene expression profiles following IL‐1β stimulation of human DuF, transcriptome sequencing of these cells was conducted in naive and IL‐1β treatment groups to identify potential molecules that mediate pro‐angiogenic action on endothelial cells. DEGs with a *p* value <0.05 and a two‐fold change in IL‐1β‐stimulated human DuF were used. In total, 497 genes were differentially expressed between naïve and IL‐1β‐stimulated DuF groups, among which 329 genes were upregulated and 168 genes were downregulated. The DEGs were biologically interpreted, and a number of proangiogenic and inflammatory genes were found to be upregulated after IL‐1β stimulation for 48 h, such as chemokine (C‐C motif) ligand (*CCL*), *IL6*, chemokine (C‐X‐C motif) ligand 8 (*CXCL8*), SPARC‐related modular calcium‐binding protein 1 (*SMOC1*), secretogranin II (*SCG2*), prostaglandin‐endoperoxide synthase 2 (*PTGS2*), and *IL1β* (Figure 1b). We focused on the eight DEGs related to proangiogenic and chemokine functions, which showed the most marked upregulation between the naive DuF and IL‐1β‐stimulated DuF groups. The upregulation of these factors after IL‐1β treatment was further determined using qRT‐PCR. Similarly, the mRNA expression data obtained through qRT‐PCR showed upregulation of all the genes and caspase‐1 (*CASP1*), which is an enzyme that converts pro‐IL‐1β to mature IL‐1β (Figure 1c).

These findings indicate that IL‐1β stimulation modulates the gene expression of proangiogenic and inflammatory mediators in DuF. This phenomenon supports the hypothesis that the inflammatory microenvironment of the dura mater during the surgical procedure may evoke the angiogenic potential of endothelial cells.

### 2.2 Potential contributing factors derived from IL‐1β‐stimulated DuF induce the gene and protein expression of proangiogenic and inflammatory factors in human microvascular endothelial cells

**FIGURE 2:**
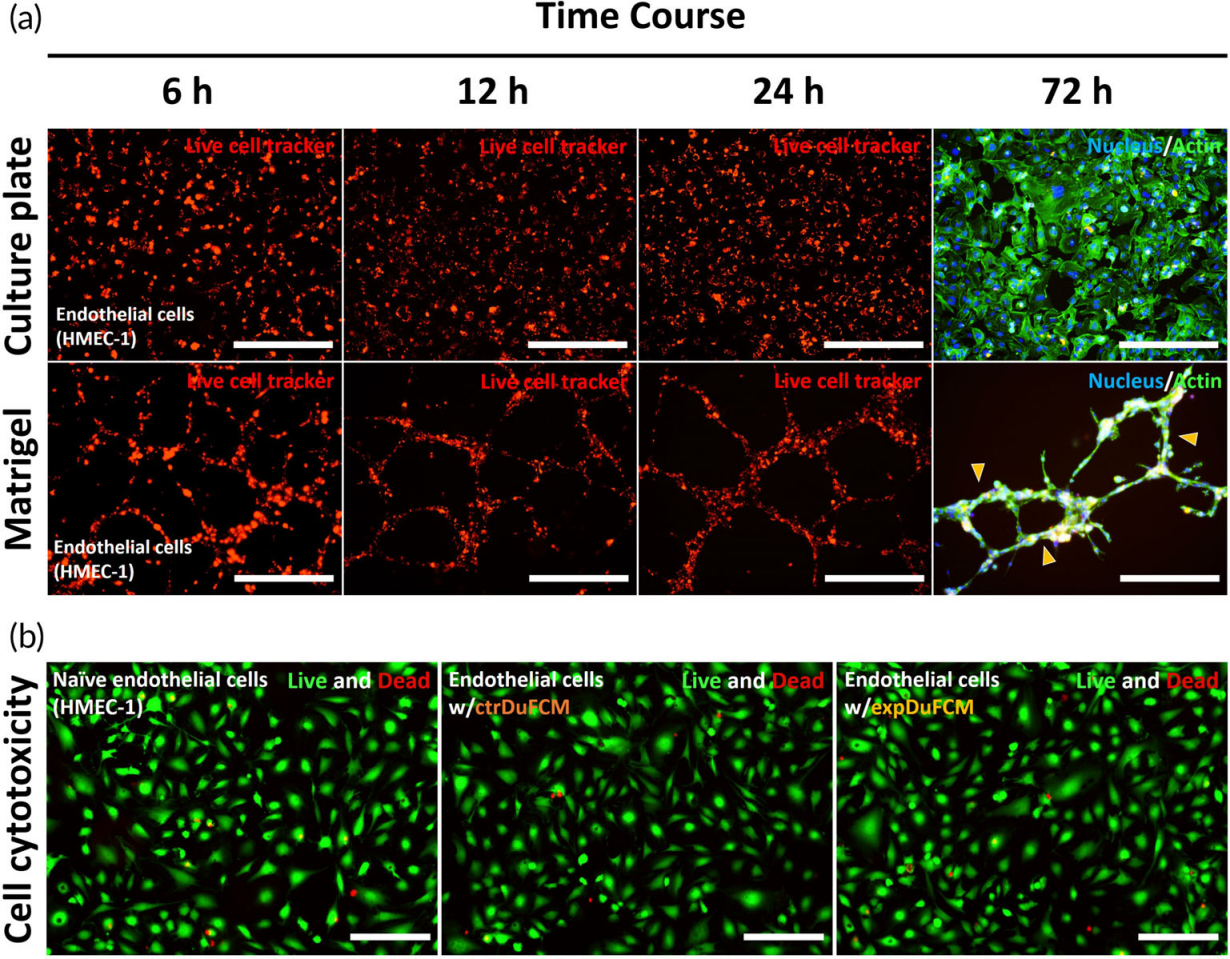
Morphological characterization of HMEC‐1 and assessment of cytotoxicity of HMEC‐1 exposed to DuFCM. (a) Fluorescence images show that HMEC‐1 cultured in Matrigel formed capillary‐like structures and branching network up to 72 h culture time (yellow arrowhead). Thus, the HMEC‐1 used in the present study exhibits the morphological characteristics of endothelial cells. (b) Assessment of cytotoxicity using live/dead assay. The results show that HMEC‐1 exposed to DuFCM did not exhibit any difference in the cell viability.

A key phenotypic feature of microvascular endothelial cells is their capacity to form capillary‐like structures. To verify that HMEC‐1, used in this study as a model endothelial cell line, maintained this feature in culture, HMEC‐1 cells were seeded on Matrigel for 72 h to observe morphological alterations using live cell tracking and immunofluorescence analysis. After 6 h, HMEC‐1 formed a cohesive branched structure. The cells continued to form capillary‐like structures, and this phenotypic alteration persisted for 72 h of culture. Thus, HMEC‐1 used in this study exhibited the characteristics of endothelial cells (Figure 2a).

Next, we investigate the expression of proangiogenic factors and chemokines in HMEC‐1 cells exposed to soluble factors derived from naïve DuF (defined as ctrDuFCM) or IL‐1β‐stimulated DuF (defined as expDuFCM). First, because treatment with DuFCM can damage HMEC‐1, we examined cytotoxicity toward HMEC‐1 in the presence of ctrDuFCM or expDuFCM using a live/dead assay. None of the groups showed changes in cytotoxicity. Hence, the data obtained using HMEC‐1 in this study are reliable (Figure 2b).

**FIGURE 3:**
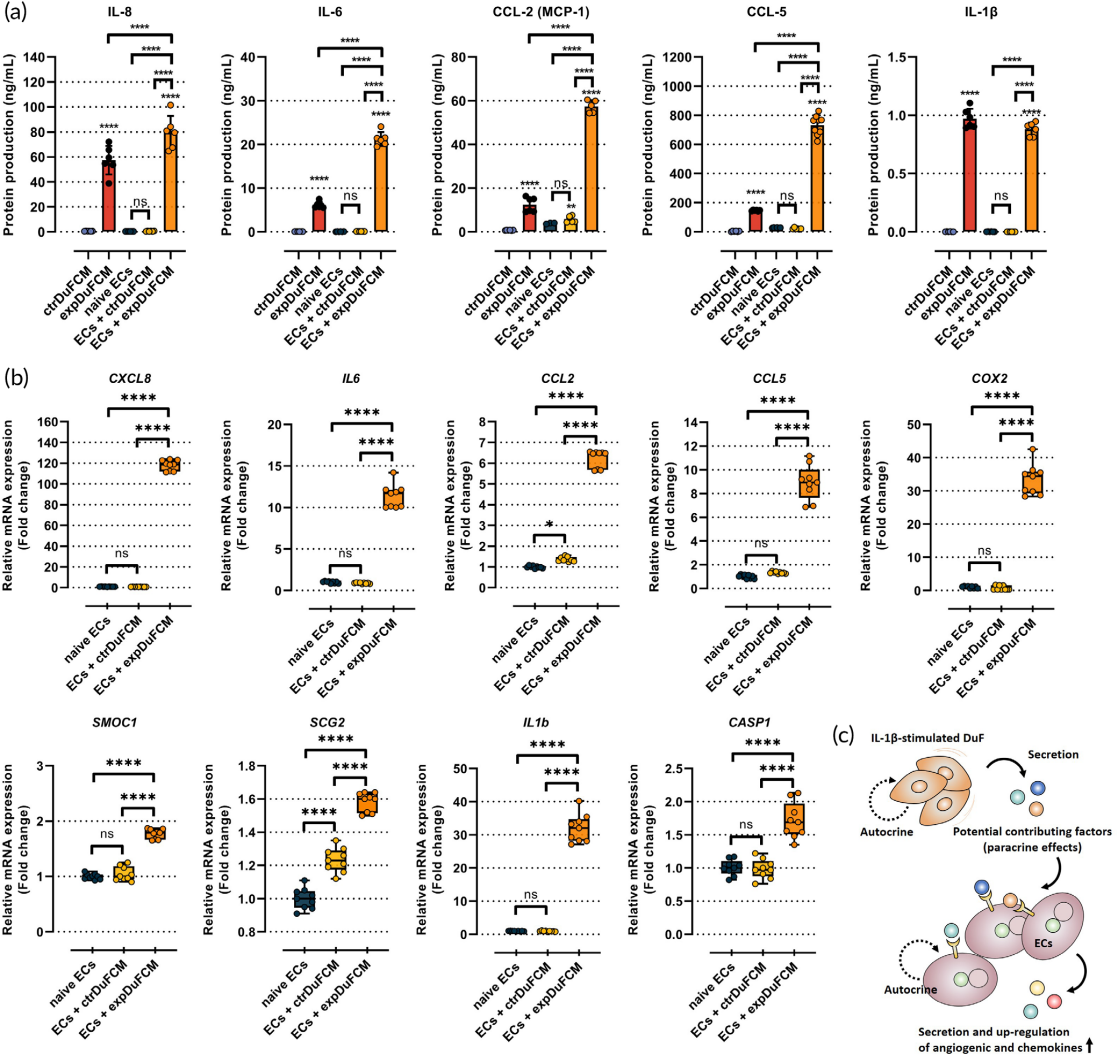
Gene and protein expression of pro‐angiogenic factors and chemokines in HMEC‐1 exposed to DuFCM. (a) Protein production and (b) gene expression of pro‐angiogenic and chemokines in HMEC‐1 exposed to DuFCM. (c) Schematic representation of the effects of soluble factors derived from DuF on angiogenic capability of HMEC‐1. The results indicate that soluble factors derived from IL‐1β‐stimulated DuF (expDuFCM) induce the gene and protein expression of pro‐angiogenic factors, chemokines, and pro‐inflammatory cytokines in HMEC‐1. The values are reported as the mean ± standard error of six or nine independent experiments. ****p < 0.0001 compared to ctrDuFCM. The line indicates the comparison with each group. ctrDuFCM, conditioned medium (CM) derived from naïve DuF; expDuFCM, CM derived from IL‐1β‐stimulated DuF; ECs, endothelial cells.

In addition, we examined the gene and protein expression of IL‐8 (CXCL8), IL‐6, CCL‐2 (MCP‐1), CCL‐5, IL‐1β, COX‐2, SMOC1, SCG2, and CASP1 using qRT‐PCR and ELISA, respectively. The experimental conditions were evaluated and divided into five groups: (1) soluble factors derived from naïve DuF (ctrDuFCM), (2) IL‐1β‐stimulated DuF (expDuFCM), (3) HMEC‐1 monoculture (naïve ECs), (4) HMEC‐1 exposed to ctrDuFCM, and (5) expDuFCM. The protein production of IL‐8, IL‐6, CCL‐2, CCL‐5, and IL‐1β, which are major secretion proteins that function as inflammatory mediators and pro‐angiogenic factors, was significantly higher in IL‐1β‐stimulated DuFs than in naive DuFs. Furthermore, the results revealed that HMEC‐1 cultured in expDuFCM induced a significantly higher production of all target proteins than that when cultured in a naive medium or ctrDuFCM. Additionally, HMEC‐1 cells exposed to expDuFCM showed higher production of these factors than those exposed to both ctrDuFCM and expDuFCM only, except for IL‐1β in expDuFCM (Figure 3a). Similar to protein production, HMEC‐1 exposed to expDuFCM exhibited significantly higher gene expression of all the aforementioned factors; proangiogenic‐related genes, including *SMOC1* and *SCG2*; and proinflammatory gene *COX2* than the naive HMEC‐1 or HMEC‐1 exposed to ctrDuFCM. Interestingly, HMEC‐1 cultured in expDuFCM also showed gene expression of *IL‐1β* and *CASP1*, which are major contributors to mature IL‐1β synthesis (Figure 3b).

These results demonstrate that the potential contributing factors derived from DuF stimulated by IL‐1β contribute to the expression of proangiogenic factors, inflammatory mediators, and chemokines in HMEC‐1, which are responsible for angiogenesis in human endothelial cells (Figure 3c).

### 2.3 Significant migration and angiogenic activity of human microvascular endothelial cells induced by soluble factors derived from IL‐1β‐stimulated DuF on a microfluidic co‐culture platform combined with 3‐dimensional ECM

**FIGURE 4:**
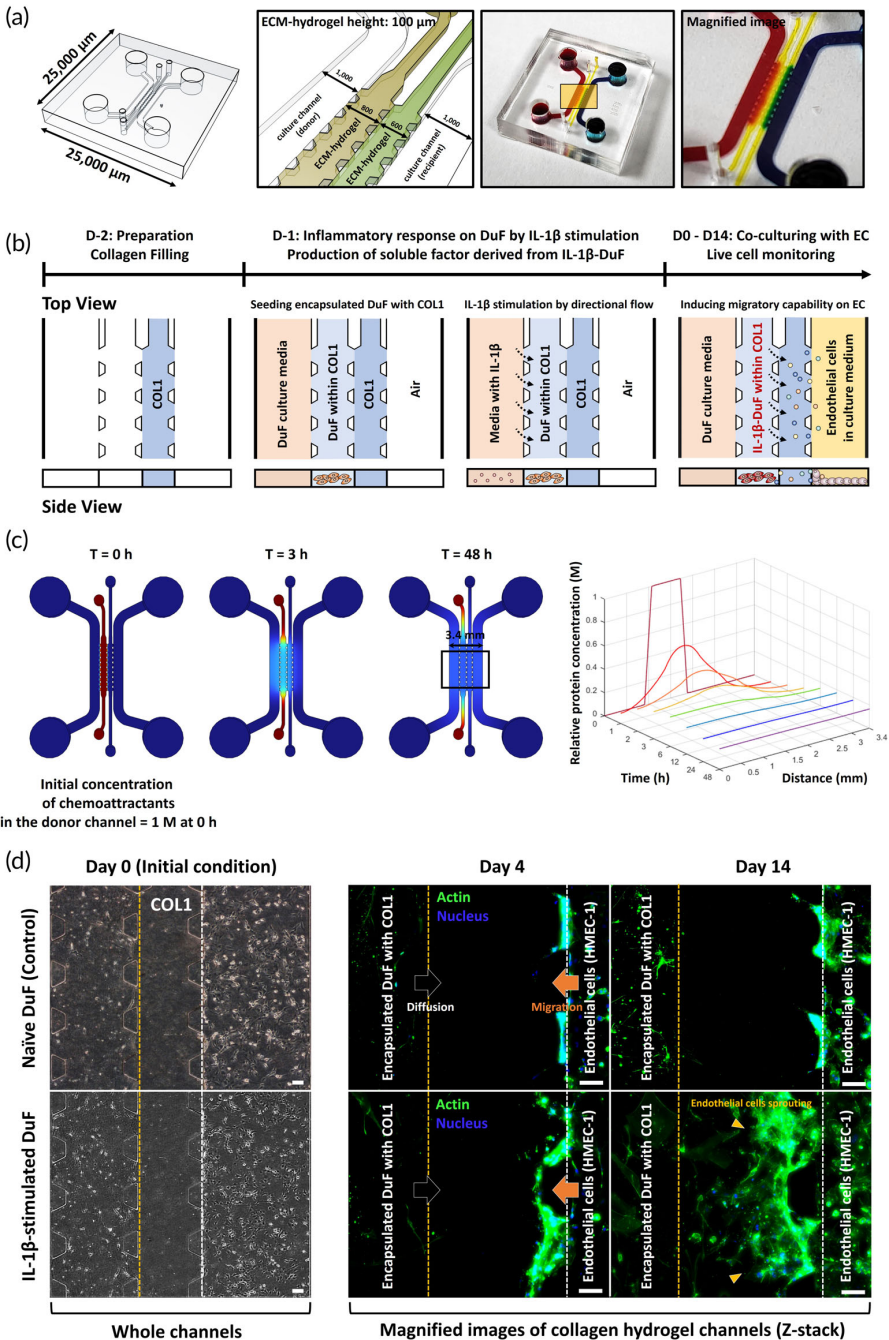
A Schematic diagram and experimental set‐up using a microfluidic platform to investigate the migration properties of ECs co‐cultured with DuF. (a) Schematic and photograph of the microfluidic device. The device consists of two culture channels and two ECM hydrogel channels. The ECM hydrogel used in this study is made of collagen type I, which is a major ECM component of dura mater. The magnified image shows the bidirectional flow between channels. (b) Schematic and description of the experimental conditions for cell‐to‐cell interaction in the microfluidic platform; all scales are represented in μm. (c) The diffusion profile of the chemo‐attractants used in this study was characterized by measuring the gradient (left) and normalized concentration plot (right) over a period of 0 to 48 h. (d) HMEC‐1 was cultured in case of co‐culture with naïve DuF (control) or IL‐1β‐stimulated DuF. The DuFs were encapsulated within a collagen hydrogel, and HMEC‐1 was attached to the surface of the collagen hydrogel (initial condition). In addition, the patterned posts on the devices allowed ECM hydrogel to align to a defined position without leakage of its materials. Fluorescence images show that the HMEC‐1 co‐cultured with IL‐1β‐stimulated DuF exhibits significant migration and invasion into the region of ECM hydrogel up to 14 days of culture time. Scale bar = 100 μm.

We developed a microfluidic coculture platform to examine the migration properties of human endothelial cells’ response to soluble factors derived from DuF (Figure 4a). We have considered the following factors for applying EDAS to microfluidics in this study: (1) the clinical evidence of angiogenic effects after the EDAS surgical procedure demonstrates the necessity of the interaction between dural fibroblasts and endothelial cells. (2) The incision of the dura mater during this process induces an inflammatory microenvironment and/or wound healing conditions within the dura mater. (3) The initiation of the inflammatory environment is mediated by pro‐inflammatory cytokines produced by resident cells within the tissue. (4) The dura mater consists of dural fibroblasts, which contribute to a microenvironment abundant in collagen type 1 expression in the extracellular matrix (ECM), while vascular endothelial cells are maintained outside the dura mater, acting as a lining. To this end, DuFs encapsulated in a 3D collagen type 1 scaffold, used as donor cells, were cultured in the donor channels. HMEC‐1 cells used as recipient cells were seeded in the recipient channels and allowed to adhere for 6 h at 37°C in a 5% CO_2_ incubator. Nonadherent cells were removed after 6 h by washing with a culture medium. Next, we monitored individual HMEC‐1 migrating to the donor channels with DuF with or without IL‐1β stimulation for 2 weeks under live cell microscopy (Figure 4b). Additionally, to eliminate any residual activity of the recombinant IL‐1β protein, we replaced the culture medium after 48 h of IL‐1β treatment and subsequently co‐cultured the endothelial cells. Prior to performing the co‐culturing experiments, we conducted a three‐dimensional simulation using computational fluid dynamics in COMSOL Multiphysics to characterize the shape of the chemical gradient resulting from the diffusion of chemoattractants secreted by the DuF into the hydrogel channel. The simulation results showed that when the concentration of chemoattractants in the donor channel, which is expected to be secreted by DuF with/without IL‐1β, was assumed to be 1 M, the molecules diffused into the recipient channel through the collagen hydrogel channel over time. After 48 h, a uniform concentration gradient was formed across all channels. The final concentration in the recipient channel was generated at approximately 0.2 M (Figure 4c).

The HMEC‐1 cells showed increased migration into 3D collagen type 1 hydrogel when soluble factors derived from IL‐1β‐stimulated DuF were introduced, whereas HMEC‐1 did not show any migration properties toward naïve DuF. These features persisted until 2 weeks of culture, and the cells simultaneously exhibited higher migratory properties (Figure 4d). These findings suggest that soluble substances from DuF stimulated by IL‐1β enhance the migratory capability of human endothelial cells through the expression of proangiogenic factors and chemokines after EDAS intervention, resulting in angiogenesis.

### 2.4 NF‐κB signaling pathway and IL‐8 mediate the IL‐1β‐DuF‐induced promotion of angiogenesis on the ECs

In this study, both DuF and ECs exhibited the highest gene and protein expression levels of IL‐8 among the chemokines and pro‐angiogenic factors secreted by IL‐1β‐stimulated DuF. We hypothesized that IL‐8 may play a significant role in endothelial cell migration and sprouting. As shown in the results, the NF‐κB signaling pathway plays an important role in the expression of IL‐8, and we treated the DuF with BAY11‐7082, an inhibitor of NF‐κB IKK protein.

**FIGURE 5:**
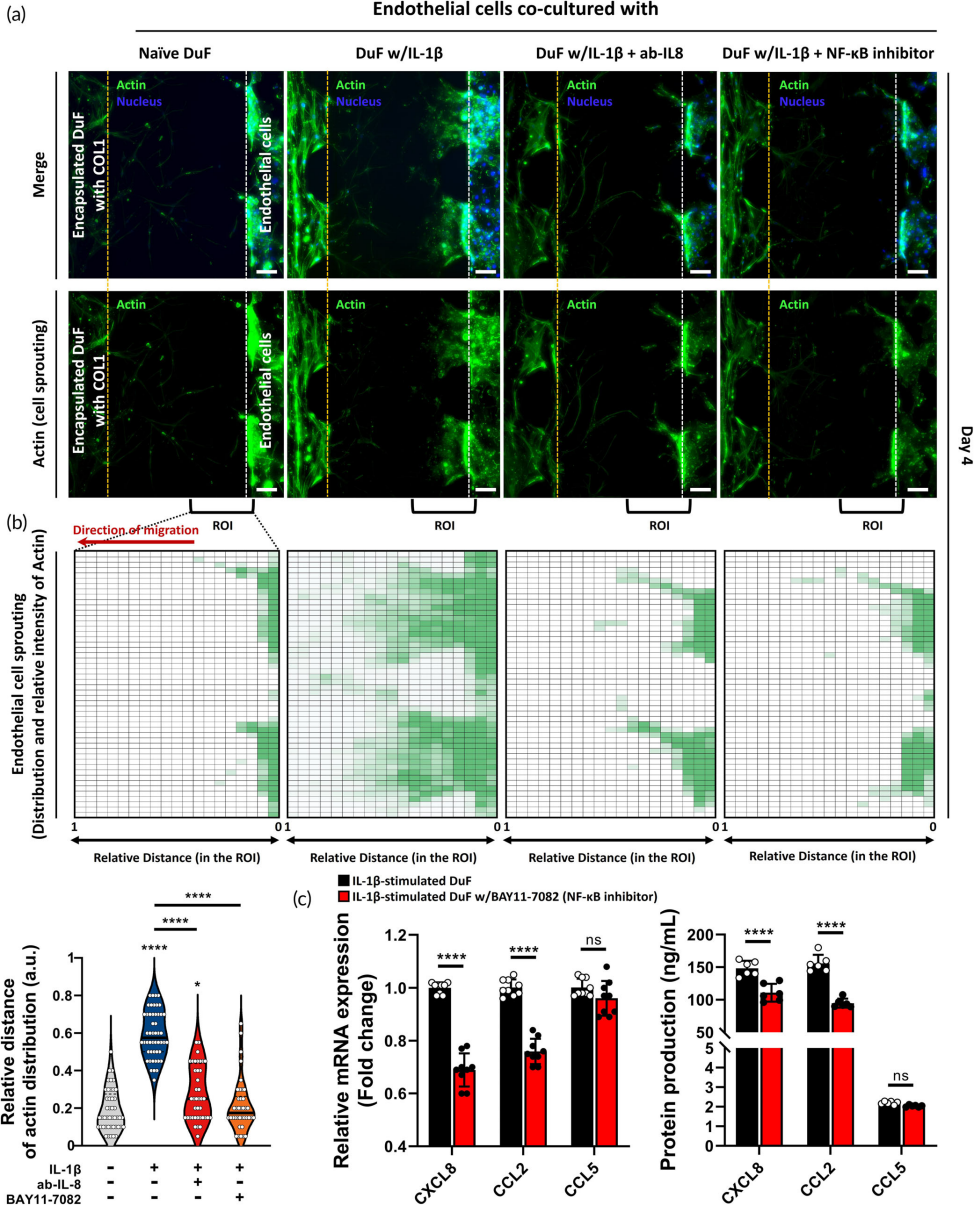
The inhibitory effects of IL‐8 neutralizing antibodies or NF‐kB signaling inhibitor on the migration properties of HMEC‐1 induced by IL‐1β‐stimulated DuF. (a) Fluorescence images and (b) Quantitative results indicate that neutralization of IL‐8 protein or NF‐κB IKK inhibitor (BAY11‐7082) reduced the chemotaxis effects on HMEC‐1 induced by soluble factors derived from IL‐1β‐stimulated DuF. The heat‐map shows the Actin distribution in the collagen‐hydrogel channels. (c) The mRNA and protein expression of major chemo‐attractants, including CXCL8, CCL2, and CCL5, by IL‐1β‐stimulated DuF with/without BAY11‐7082. ****p < 0.0001 compared to the naïve DuF co‐culturing group. The line indicates the comparison with each group. Scale bar = 100 μm.

To test our hypothesis, we used the microfluidic platform to co‐culture ECs with IL‐1β‐stimulated DuF, supplemented with IL‐8 neutralizing antibodies or BAY11‐7082. The immunofluorescence images (Figure 5a) and quantitative results (Figure 5b) showed that both neutralization of IL‐8 protein and inhibition of NF‐κB signaling significantly reduced HMEC‐1 migration and sprouting toward the collagen‐hydrogel compartment compared with the co‐culturing IL‐1β‐stimulated DuF group. In particular, the ECs treated with BAY11‐7082 showed a tendency to completely inhibit the actin distribution induced by soluble factors derived from IL‐1β‐stimulated DuF, with no significant difference compared to those of the naïve DuF group. Furthermore, the treatment of ECs with BAY11‐7082 was able to attenuate the gene and protein expression levels of both IL‐8 and CCL‐2 (Figure 5c).

Our findings suggest that soluble substances from DuF stimulated by IL‐1β enhance the migratory capability of human endothelial cells through the expression of proangiogenic factors and chemokines after EDAS intervention, resulting in angiogenesis. Specifically, IL‐8 secreted by IL‐1β‐stimulated DuF via NF‐κB signaling pathway can be the major chemoattractant for endothelial migration and sprouting.

## 3 DISCUSSION

**FIGURE 6:**
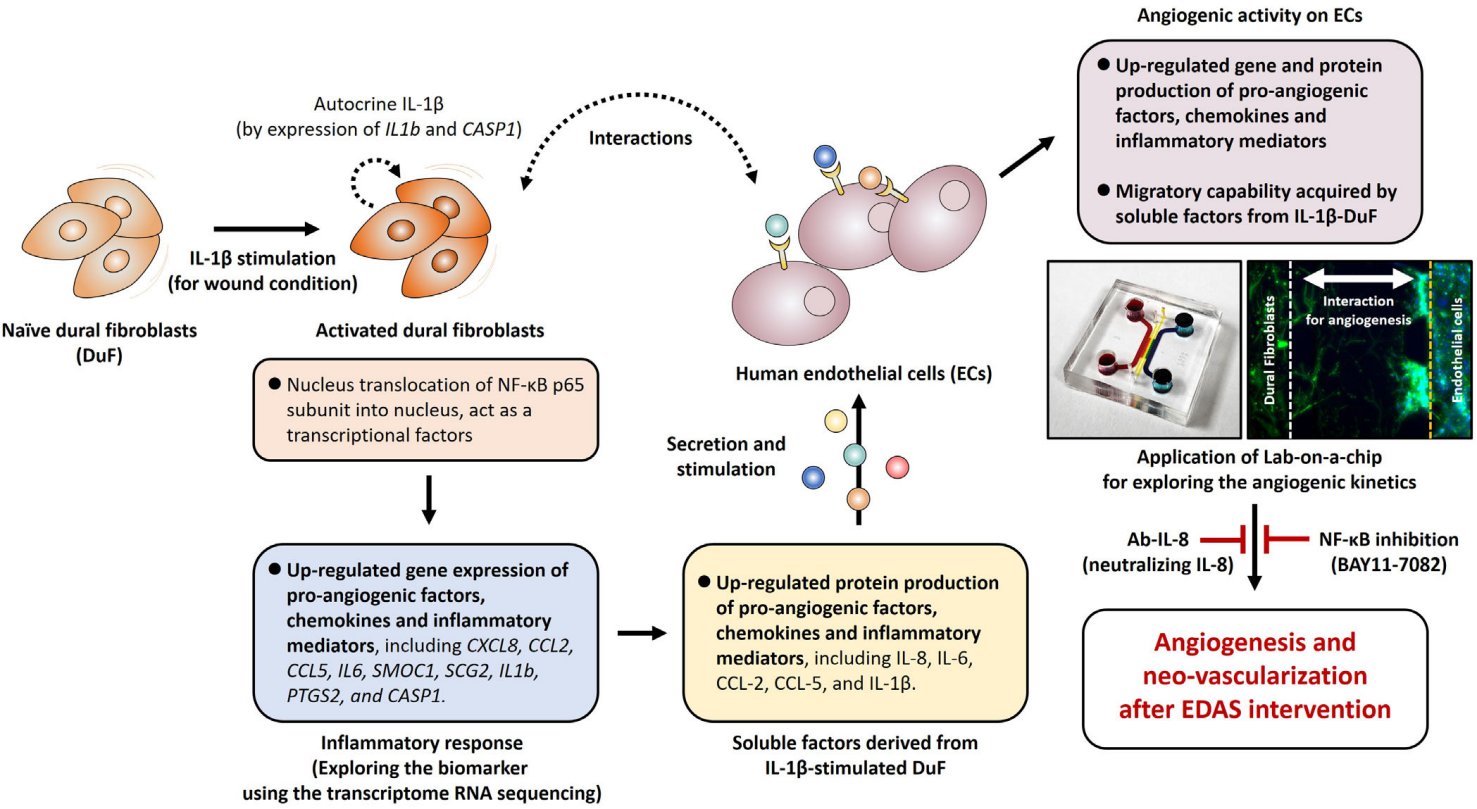
Schematic summary of the EDAS in vitro model and the effects of soluble factors derived from DuF on the endothelial cell's activation and migration for angiogenesis.

Indirect revascularization, such as EDAS, is performed by transplanting the extracranial vessel and the vascularized dura mater directly onto the brain and target neovascularization to the underlying brain cortex. Indirect revascularization is already widely accepted as a primary treatment of MMD for improving collateral blood flow and secondary stroke prevention in patients with MMD, and given the poor clinical results of non‐surgical treatment and the relatively good outcome of surgery, it is consistently gaining popularity.^5^, ^20^, ^22^ Numerous clinical studies have been introduced, reporting good results in terms of safety profile, stroke prevention, and overall outcomes.^22^, ^23^ However surprisingly little is known about the molecular mechanisms underlying this phenomenon and the interaction between the transplanted donor tissue/vessels and the recipient brain cortex. It is obvious that certain interactions between DuF and endothelial cells will be triggered after surgical intervention, and this interaction can probably act as an important step for neovascularization. Our study provides the first evidence that soluble factors derived from DuF stimulated by IL‐1β enhance the production and expression of proangiogenic factors and chemokines and induce the migratory capability of human endothelial cells, which is expected to occur during the process of neovascularization after surgical intervention. This result supports the idea that DuF‐endothelial cell interaction under inflammatory response can play a key role in intracranial neovascularization. This is a very new experimental in vitro evidence supporting the utilization of indirect revascularization for MMDs (Figure 6).

Inflammatory reaction is not made intentionally, however, is inevitable as it is a subsequent reaction following surgical intervention and related injuries to the intracranial tissues. IL‐1β is a major regulator of various cellular responses, including wound healing, inflammation, ECM remodeling, and angiogenesis, which in turn lead to the biological microenvironment and phenotypic changes in diverse tissues or cells.^19^, ^24^ This proinflammatory cytokine has a number of functions, including chemotaxis of immune cells, adhesion molecule expression on endothelial cells, and induction of inflammatory mediators, chemokines, and proangiogenic factors on fibroblasts.^25^, ^26^ After tissue injury or surgical incision, acute inflammation occurs during the early stages of wound healing. This process involves resident fibroblasts and immune cells that secrete a variety of soluble factors responsible for maintaining tissue homeostasis.^12^, ^16^ The primary resident cell type in the meninges, particularly the dura mater, is dural fibroblasts. These cells maintain the ECM of the dura mater by producing collagen and fibronectin. In addition, the cells control the inflammatory milieu via the release of inflammatory substances into adjacent cells.^12^, ^27^, ^28^ Taken together, the aforementioned roles of fibroblasts in tissues suggest that during the EDAS procedure, surgical incision injury of the dura mater might lead to an iatrogenic inflammatory environment, and subsequent upregulation of the expression of IL‐1β is expected. Furthermore, this phenomenon hypothetically may upregulate the secretion of inflammatory mediators, including chemokines, proinflammatory cytokines, and proangiogenic factors by dural fibroblasts, resulting in the enhancement of angiogenesis. Indeed, in response to a wound in the brain, upregulated expression of IL‐1 has been observed and is known to stimulate neovascularization.^29^, ^30^

In the present study, we treated IL‐1β on human DuF, as an incisional injury in vitro model of dura mater during EDAS. Our results showed that IL‐β stimulation induces gene and protein expression of a variety of mediators in DuF, including chemokines and proangiogenic factors. Interestingly, the cells also expressed the gene and protein of IL‐1β and induction of endogenous CASP‐1, which mediate autocrine signaling in DuF. IL‐1 is generally synthesized as a precursor protein (pro‐IL‐1β) that is biologically inactive. This precursor is proteolytically cleaved by CASP‐1 to produce its active form in the cytoplasm. In addition, IL‐1β has been closely linked to the activation of the NF‐κB signaling pathway via nuclear translocation of the NF‐κB subunits (p65 and p50 intracellular protein), inducing the inflammatory and pro‐angiogenic gene encoding.^31^, ^32^ Similarly, our immunofluorescence data revealed that IL‐1β‐stimulated DuF exhibits translocation of p65 protein into the nucleus, acting as a transcriptional factor. In addition to the inflammatory reaction to IL‐1β, the DuF cells showed gene and protein production of proangiogenic factors, chemokines, and inflammatory mediators, including IL‐8, IL‐6, CCLs, COX2, SMOC1, and SCG2. IL‐8, a chemokine with defining CXC amino acid motif, is known to regulate angiogenesis by modulating endothelial proliferation and survival.^33^, ^34^ IL‐6, a proangiogenic factor, has been shown to influence angiogenesis.^35^, ^36^ A study has shown that the overexpression of IL‐6 in transgenic mice induces hypervascularization of the cerebellum and enhances the production of vascular endothelial growth factor (VEGF) by tumor cells.^36^ CCLs, a family of chemokines with two adjacent cysteines (CC motif), include CCL‐2 and CCL‐5. CCLs, including CCL‐1, CCL‐2, and CCL‐5, are involved in inflammation‐mediated angiogenesis. In particular, some studies have demonstrated that both CCL‐2 and CCL‐5 are associated with endothelial cell migration, neovascularization, and tubule formation during angiogenesis.^37^, ^38^ It has also been reported that the upregulation of SMOC1, a matricellular protein, increases endothelial cell proliferation and tube formation. Furthermore, the authors demonstrated that silencing SMOC1 by treatment with small interfering RNA (siRNA) attenuated endothelial cell migration, proliferation, and sprouting in angiogenesis assays.^39^, ^40^ The SCG2 gene encodes the protein secretogranin, which is rapidly cleaved into bioactive secretoneurin. This active protein is involved in the wound‐healing process and has been shown to enhance the proliferation, migration, and angiogenesis of endothelial cells.^40^ The enhanced production and expression of these pro‐inflammatory, pro‐angiogenic molecules by DuF indicates the upregulated angiogenesis under inflammatory reactions. This finding is compatible with previous studies revealing the promotion of angiogenesis after wound healing or inflammations. Our results suggest the significance of DuF‐endothelial cell reaction under pro‐inflammatory stimulation, and the increased expression/production of angiogenesis‐related factors support the idea that this phenomenon might play a key role during intracranial neovascularization.

Consistent with the aforementioned findings, we found that soluble factors derived from IL‐1β‐stimulated DuF markedly enhanced gene and protein production of these factors in human endothelial cells. Furthermore, human endothelial cells cocultured with IL‐1β‐stimulated DuFs exhibit considerable migratory properties in a microfluidic coculture platform. This result can be considered as a solid evidence that DuF‐endothelial cell interaction results in enhanced migration of endothelial cells, which is a critical step in neovascularization. Taken together, these findings demonstrate that soluble substances from DuF may induce angiogenesis in human endothelial cells via secretion and upregulation of proangiogenic factors and chemokines through cell‐to‐cell interactions after EDAS intervention. The conclusions were in good agreement with previous literature, but the current study has some limitations. First, because angiogenesis by endothelial cells is strongly associated with ECM components for biological signals, it is important to include various ECM components such as fibronectin, laminin, and other types of collagens within the hydrogel structure of the microfluidic platform. With regard to this, ECM‐modifying enzymes such as matrix metalloproteinases (MMPs) are also necessary for the degradation of the basement membrane during angiogenesis. This study focused primarily on gene and protein production related to inflammatory/angiogenic mediators, chemokines, and direct proangiogenic factors, and further experimental studies including the ECM regulatory mechanism should be added. Another limitation is that based on our current results, we have discovered several key molecules that might be involved in the inflammatory reaction or the angiogenesis, but this does not necessarily represent the actual in vivo environment and we still do not know the detailed downstream molecular mechanism after EDAS.

### 3.1 Materials and Methods

#### 3.1.1 Primary human DuF culture and IL‐1β stimulation for inflammatory conditions

Primary human DuFs, which were isolated from a 20‐week‐old male fetus, were purchased from ScienCell (Carlsbad, CA, USA) and cultured according to the manufacturer's instructions. Cells were cultured in a fibroblast medium (ScienCell, Carlsbad, CA, USA) supplemented with 10% fetal bovine serum (FBS; ScienCell, Carlsbad, CA, USA), 1% fibroblast growth supplement (ScienCell, Carlsbad, CA, USA), and 1% penicillin/streptomycin (P/S; ScienCell, Carlsbad, CA, USA). After subculturing the DuFs, the culture medium was replaced with Dulbecco's modified Eagle's medium (DMEM)/F12 containing 1% P/S and 1% FBS, with or without recombinant human IL‐1β (1 ng/mL) for 48 h. Medium collected from human DuF culture is referred to as “ctrDuFCM” (without IL‐1β) or “expDuFCM” (with IL‐1β).

#### 3.1.2 Immortalized human microvascular endothelial cell (HMEC‐1) culture

The immortalized HMEC‐1 cells, which were isolated from the endothelium of the foreskin of a male patient, were cultured in MCDB 131 medium (Sigma‐Aldrich, Burlington, MA, USA) supplemented with 10% FBS (ATCC, Manassas, VA, USA), P/S (ScienCell, Carlsbad, CA, USA), 10 ng/mL epidermal growth factor (Sigma‐Aldrich, Burlington, MA, USA), 1 μg/mL hydrocortisone, and 2 mM L‐glutamate (Thermo Fisher Scientific, Waltham, MA, USA) in 75 cm^2^ culture flasks as a control group. At approximately 80% confluency of HMEC‐1, 5.0 × 10^5^ cells were subcultured in a 75 cm^2^ cell culture flask. The supernatant was collected and stored at −80°C for enzyme‐linked immunosorbent assay (ELISA).

#### 3.1.3 Culturing of HMEC‐1 in the conditioned medium collected from human DuF culture

HMEC‐1 cells were cultured in DMEM/F12 supplemented with 1% FBS and 1% P/S at a density of 5.0 × 10^5^ cells in a 25 cm^2^ cell culture flask for 48 h as a control group. In addition, HMEC‐1 cells were cultured in ctrDuFCM and expDuFCM for 48 h as the experimental groups. The supernatants and mRNA extract were collected and stored at −80°C for ELISA.

#### 3.1.4 Gene expression profile analysis

The total RNA was isolated using TRIzol reagent. The RNA quality was assessed using an Agilent 2100 bioanalyzer with an RNA 6000 Nano Chip (Agilent Technologies, Santa Clara, CA, USA), and RNA quantification was performed using an ND‐2000 spectrophotometer (Thermo Fisher Scientific, Waltham, MA, USA). For control and test RNAs, library construction was performed using the QuantSeq 3′ mRNA‐Seq Library Prep Kit (Lexogen, Vienna, Austria) according to the manufacturer's instructions. High‐throughput sequencing was performed as single‐end 75 sequencing using a NextSeq 500 (Illumina, San Diego, CA, USA). QuantSeq 3′ mRNA‐Seq reads were aligned using Bowtie2 (Langmead and Salzberg, 2012). Bowtie2 indices were generated from either the genome assembly sequence or representative transcript sequences for alignment to the genome and transcriptome. The alignment file was used to assemble transcripts, estimate their abundance, and detect the differential expression of genes. Differentially expressed genes (DEGs) were determined based on counts from unique and multiple alignments using Bedtools (Quinlan AR, 2010). The read count data were processed based on the TMM + CPM normalization method using EdgeR within R (R Development Core Team, 2020) and Bioconductor (Gentleman et al., 2004). Gene classification was based on searches performed using DAVID (http://david.abcc.ncifcrf.gov/) and MEDLINE (http://www.ncbi.nlm.nih.gov/) databases. The raw RNA‐seq data files were deposited in NCBI's Gene Expression Omnibus (GEO) and are accessible via the GEO Series accession number GSE224999.

#### 3.1.5 Immunofluorescence staining of nuclear factor kappa‐light‐chain‐enhancer of activated B cells (NF‐κB) p65

To localize p65 protein expression in human DuFs, cells were plated on a glass‐bottom confocal dish and exposed to IL‐1β (10 ng/mL) for 48 h. The disk cells were fixed with 4% paraformaldehyde and permeabilized with 0.2% Triton X‐100 in PBS for 10 min at 25°C. The cells were blocked with 5% bovine serum albumin (Merck Millipore, Burlington, MA, USA) in PBS and incubated with the primary antibody against NF‐κB p65 (1:100; Sigma‐Aldrich, Burlington, MA, USA), followed by incubation with Alexa 555 secondary antibodies (1:200; Invitrogen, Waltham, MA, USA). The samples were imaged using an EVOS FL auto cell imaging system (Thermo Fisher Scientific, Waltham, MA, USA).

#### 3.1.6 Enzyme linked immunosorbent assay (ELISA)

The concentrations of IL‐8, IL‐6, CCL‐2 (MCP‐1), CCL‐5, and IL‐1β in the supernatant were measured using commercially available ELISA kits (R&D Systems, Minneapolis, MN, USA) according to the manufacturer's instructions.

#### 3.1.7 Quantitative real‐time polymerase chain reaction (qRT‐PCR)

HMEC‐1 and human DuFs were lysed using TRIzol reagent, RNA was extracted, and cDNA was synthesized according to the manufacturer's instructions. qRT‐PCR was performed to determine the mRNA levels of *CXCL8, IL6, CCL2, CCL5, PTGS2, SMOC1, SCG2, IL1β*, and *CASP1* using the SYBR Green PCR Master Mix (Applied Biosystems, Waltham, MA, USA). The mRNA expression was analyzed using the 2−∆∆Ct method.

#### 3.1.8 Cell cytotoxicity (live/dead assay)

HMEC‐1 cells were plated in a 25 cm^2^ culture flask and treated with DMEM/F12, ctrDuFCM, and expDuFCM for 48 h. After receiving different treatments at the indicated times, the culture media were replaced with fresh media supplemented with live/dead reagents, according to the manufacturer's instructions.

#### 3.1.9 Fabrication of chemotaxis platform and migration test

The microfluidic chemotaxis platform was fabricated from polydimethylsiloxane (PDMS; Dow Corning, Midland, MI, USA) using standard photolithography. A mixture of a PDMS base and curing agent in a 9:1 ratio was poured onto the master mold and cured at 70°C in a dry oven for at least 2 h. The PDMS was then detached from the master mold and punched into the reservoirs. After that, it was bonded to a coverslip using a plasma generator (FEMTO SCIENCE, Hwaseong, Gyeonggi, Korea). The devices were autoclaved at 121°C for 15 min and stored in a dry oven to restore hydrophobicity before use. We used the microfluidic chemotaxis platform to investigate the interaction between DuF and ECs. The four distinct chambers of the microfluidic platform had cell culture chambers that could incubate different cells, and two collagen‐hydrogel chambers. Multiple posts were placed in the collagen‐hydrogel chambers to create a three‐dimensional hydrogel scaffold for gel injections. The cell seeding protocols for the interaction between DuF and ECs are illustrated in Figure 4b.

#### 3.1.10 COMSOL simulation

We utilized COMSOL Multiphysics (COMSOL, Burlington, MA, USA) to establish a microenvironment model for simulating the diffusion coefficient of chemokines with a molecular weight of 11 kDa (average molecular weight of chemokines) in type 1 collagen at 37°C. The diffusion simulation was calculated in the x–y plane, where the chemokines diffused through the connected channels with any external flux. We set the diffusion coefficient to 5.8 × 10^−11^ m^2^/s in a collagen‐hydrogel (2.0 mg/mL), and the generation concentration of chemokines was set to a uniform concentration of 1 M in the DuF‐encapsulated hydrogel channel. The pressure and concentration in the other channels were set to 0.

#### 3.1.11 Statistical analysis

Data were expressed as the mean ± standard deviation of four individual experiments using independent cell cultures. One‐way analysis of variance and Bonferroni's correction *post‐hoc* test were used to assess the differences among the experimental groups. Statistical analyses were performed using GraphPad Prism 9 (GraphPad Software, San Diego, CA, USA). Statistical significance was set at *p* < 0.05.

## 4 CONCLUSIONS

This study represents an important advance in our knowledge of the molecular mechanism of the interaction between DuF and endothelial cells that are expected to occur during angiogenesis after EDAS intervention. An enhanced understanding of the contributors such as the major molecules of angiogenesis could enable the identification of novel therapeutic targets and the effective treatment of MMD through enhancing the angiogenesis. Once we unveil the knowledge regarding the interaction between dura mater, intracranial structure and vessels, we will be able to discover possible therapeutic technologies which may enhance angiogenesis, finally maximizing the therapeutic effect of EDAS in the future. In addition, with high‐throughput and regulated microenvironments, the microfluidic platform can be extended to the study of motility or activation of other cells such as glial cells or neurons relevant to the EDAS or MMD.

## AUTHOR CONTRIBUTIONS

**Woo‐Keun Kwon:** Conceptualization (lead); data curation (lead); funding acquisition (lead); investigation (lead); project administration (lead); resources (lead); supervision (lead); visualization (lead); writing – original draft (lead); writing – review and editing (lead). **Chang‐Min Yoo:** Conceptualization (lead); data curation (lead); formal analysis (lead); investigation (lead); methodology (lead); software (lead); validation (lead); visualization (lead). **Jang Hun Kim:** Data curation (equal); writing – review and editing (equal). **Tae‐Won Kim:** Validation (equal). **An‐Gi Kim:** Validation (equal). **Min‐Ho Hwang:** Conceptualization (lead); data curation (lead); formal analysis (lead); funding acquisition (lead); investigation (lead); methodology (lead); resources (lead); software (lead); supervision (lead); validation (lead); visualization (lead); writing – original draft (lead); writing – review and editing (lead). **Hyuk Choi:** Conceptualization (lead); data curation (lead); funding acquisition (lead); investigation (lead); project administration (lead); resources (lead); supervision (lead); visualization (lead); writing – original draft (lead); writing – review and editing (lead).

## CONFLICT OF INTEREST STATEMENT

The authors have declared no conflict of interest.

### PEER REVIEW

The peer review history for this article is available at https://www.webofscience.com/api/gateway/wos/peer‐review/10.1002/btm2.10589.

## Data Availability

The datasets generated and/or analyzed during the current study are available from the corresponding author upon reasonable request.
